# Impact of corn silage substitution for dry alfalfa on milk fatty acid profile, nitrogen utilization, plasma biochemical markers, rumen fermentation, and antioxidant capacity in Mahabadi lactating goats

**DOI:** 10.1016/j.vas.2023.100323

**Published:** 2023-11-13

**Authors:** Shohre Tarverdi Sarabi, Amir Fattah, Nader Papi, Sayyed Roohollah Ebrahimi Mahmoudabad

**Affiliations:** a Department of Animal Science, Shahr-e-Qods Branch, Islamic Azad University, Tehran 3754113115, Iran; b Agricultural Research, Education and Extension Organization, Animal Science Research Institute of Iran, Karaj 3146618361, Iran

**Keywords:** Corn silage, Milk component, Antioxidant capacity, Mahabadi goats

## Abstract

This study aimed to investigate the effects of substituting conventional dry forage with corn silage, a more cost-effective supplementary feed, on a variety of parameters in lactating Mahabadi goats, native to arid and semi-arid regions. A total of fifty lactating Mahabadi goats, aged between 2 and 5 years and weighing an average of 45.3 ± 7.20 kg, were randomly allocated into two experimental groups. The experimental treatments consisted of a control diet and a diet incorporating corn silage at 20 % of dry matter (DM). Various measurements were conducted to assess milk compositions, fatty acid profiles, rumen fermentation parameters, selected plasma biochemical parameters, nitrogen balance, and antioxidant capacity. The results revealed that the substitution of dry forage with corn silage had no statistically significant impact on milk yield, dry matter intake (DMI), body weight, as well as the proportions of monounsaturated and unsaturated fatty acids, and specifically C18:1C9 in milk, non-esterified fatty acid content in milk, rumen concentration of volatile fatty acids (VFA), microbial nitrogen, and nitrogen balance (*p* > 0.05). Nonetheless, this substitution led to a reduction in the percentage of milk polyunsaturated fatty acids and plasma urea nitrogen content (*p* < 0.05). It is noteworthy that goats fed with corn silage exhibited a notably higher antioxidant capacity in rumen liquor (*p* < 0.05), plasma, and milk (*p* < 0.01) compared to those receiving the control diet. The substitution of conventional dry forage with corn silage exhibited no adverse effects on the performance of lactating Mahabadi goats. On the contrary, it contributed to an enhancement in the antioxidant status. These findings offer valuable insights for optimizing dietary composition by incorporating corn silage at 20 % of DM, thereby improving milk quality, reducing costs, and promoting overall health in lactating goats, particularly within arid and semi-arid regions.

## Highlights

• Corn silage substitution in goat diet did not impact milk yield or body weight.
• Milk fatty acid profile remained stable, except for reduced polyunsaturated fatty acids.
• Rumen fermentation parameters and nitrogen balance were unaffected by corn silage.
• Corn silage improved antioxidant capacity in rumen, plasma, and milk.
• Findings suggest corn silage can enhance milk quality and goat health without performance decline.

## 1 Introduction

In ruminant livestock production systems, the pivotal role of forage intake cannot be overstated, accounting for a substantial portion of their dietary requirements, ranging from approximately 40 to 90 % (Mahanna & Chase, 2003). The maintenance of optimal health and milk production in ruminants hinges on the stability of the rumen environment, a feat accomplished through the ingestion of an adequate quantity of forage. A decline in the intake of fibrous feedstuffs, notably those rich in Neutral Detergent Fiber (NDF), precipitates a reduction in ruminal pH levels, leading to the onset of digestive disorders (Pereira et al., 2023). This, in turn, results in diminished milk yield, dry matter intake, milk fat content, and ruminal fiber digestibility (Plaizier et al., 2008). While the consumption of fiber-rich forage is indispensable for optimizing milk yield and ensuring the health of ruminants, it concurrently exacerbates methane emissions (van Gastelen et al., 2015). Hence, the utilization of starch-rich forage presents a pragmatic approach to mitigating methane production and associated emissions (Hassanat et al., 2013).

Furthermore, the contemporary backdrop of global climate change, characterized by dwindling precipitation levels, particularly in arid and semi-arid regions, underscores the imperative need for forage crops that can thrive in drier conditions, as compared to traditional forages. Notably, in recent years, agricultural croplands have accounted for a substantial share, encompassing 70–90 %, of total global freshwater usage by humans (Foley et al., 2020). Livestock production systems similarly claim a significant slice of this agricultural water allocation (Pathak et al., 2018). Against this backdrop, the establishment of sustainable forage production systems, which are both high-yielding and high-quality, assumes paramount significance in the context of the burgeoning animal husbandry sector (Xu et al., 2021).

The preparation of silage, a conventional method for preserving forage with elevated moisture content, centers on the conversion of water-soluble carbohydrates into organic acids by lactic acid bacteria through anaerobic fermentation of various green fodder components, such as corn plant stalks, leaves, and cobs. This process yields a feed that can be stored for extended durations, characterized by a high energy content (Du et al., 2023). Consequently, the reduced rainfall experienced over the past decade has led to diminished forage production in semi-tropical regions like Iran. Given the inadequacy of Iran's grassland resources and stocking capacity, there has been a notable surge in the importation of substantial quantities of dry forage to compensate for the dearth of high-quality alternatives. Taking cost considerations into account, the utilization of corn silage emerges as a more pragmatic choice for production and utilization in arid or semi-arid regions, particularly in Iran (Taravat et al., 2017).

While the provision of high-quality feed remains essential for sustaining high milk yields, the development and utilization of low- and medium-quality forage cannot be discounted, particularly in the context of animals with lower and moderate production outputs, which have less stringent nutrient requirements. The judicious utilization of low-quality forage, following appropriate treatment, holds the potential for significant reductions in feed costs without compromising production performance. Furthermore, this approach opens new avenues for feeding goats with low-quality forage, thereby reducing feed expenses without detrimental effects on milk production. Scientific investigations have elucidated that the prudent utilization and processing of forage can curtail concentrate consumption and feeding costs, thereby contributing to extending the productive and reproductive lifespan of animals (Guo et al., 2022). Therefore, the optimization of feed composition, with a specific focus on the forage component, assumes paramount importance in enhancing dairy production efficiently and economically over the long term. In numerous regions, alfalfa and corn silage (CS) represent the prevailing choices for feeding high-producing dairy goats (NRC, 2007). The high digestibility of the fiber in these forages can alleviate gut fill, facilitating increased feed consumption and enhanced milk production (Wang et al., 2021).

Corn silage, characterized by its high starch content (25–35 % on a dry matter basis; NRC, 2007), constitutes a significant component of ruminant diets. It serves as a valuable source of fermentable energy for ruminal microflora, consequently leading to increased milk yield, higher protein concentration, and enhanced feed intake (Khaing et al., 2015). Furthermore, corn silage has been demonstrated to effectively mitigate methane production and emissions (Khan et al., 2012), suggesting that animals fed with grass-based diets are more likely to produce higher methane emissions. There are three plausible mechanisms through which corn silage can reduce methane production within the rumen. First, its elevated starch content promotes the production of propionate over acetate. Second, the increased total dry matter intake (DMI) and enhanced passage rate result in reduced ruminal residence time, leading to decreased ruminal fermentation and an increase in post-ruminal digestion. Third, the substitution of grass with corn silage enhances animal performance, leading to lower methane emissions per unit of animal product (O'Mara et al., 1998). Recent studies have consistently demonstrated the positive impacts of replacing grass with corn silage. For instance, Hassanat et al. (Hassanat et al., 2013) reported decreased methane emissions when 100 % corn silage replaced alfalfa silage. Additionally, corn silage harvested during later stages of maturity has been claimed to further reduce methane emissions (Tamminga et al., 2007). As well, its high content of polyunsaturated fatty acids (PUFA), specifically n-3 and n-6 FA, can modulate milk fatty acid profiles (Dewhurst et al., 2006). Additionally, corn silage lends itself to easy crushing, storage, and integration into ration diets compared to other forages (Neylon & Kung, 2003). Notably, corn silage also boasts a high anthocyanin content, which remains preserved during circulation and is subsequently absorbed into the milk. Anthocyanin plays a pivotal role in scavenging free radicals and augmenting antioxidant capacity (Canuto et al., 2016). However, it is imperative to take into account factors that influence corn silage quality, including silage pH levels, moisture content, aerobic decay, and forage components, as mycotoxin production during silage preparation can give rise to concerns, such as abortion in dairy cows (Schmidt et al., 2015).

In the Iranian context, governmental policies have stimulated a growing interest in the rearing of lactating goats, particularly the Mahabadi breed, distinguished by its high milk production (1.3 kg per day), twinning percentage (60–80 %), and adaptability to diverse environmental conditions (Nazemi Karkaj et al., 2021). Despite the widespread adoption of new technology for silage production in the dairy industry, its application remains relatively limited in the sheep and goat industries, despite the large population of over 65 million head in Iran (Agricultural Statistics of Iran, 2017).

Given the considerable costs associated with dry forage production and storage in Iran, silage-making has emerged as an efficient strategy. In this context, our hypothesis posits that the inclusion of corn silage, abundant in starch, PUFA, and anthocyanin, could exert a positive influence on the performance of lactating goats. Consequently, the objective of this study is to assess the impacts of substituting dry forage (dry alfalfa) with corn silage on milk yield, its components, fatty acid profiles, selected plasma biochemical parameters, rumen fermentation parameters, nitrogen balance, and antioxidant potential in Mahabadi lactating goats.

## 2 Materials and methods

### 2.1 Ethical considerations

All animal procedures were conducted in compliance with the guidelines set forth by the Animal Care and Use Committee of the Animal Science Research Institute of Iran, following the standards outlined in the Iranian Council of Animal Care (PN 2022-015A).

### 2.2 Animals, experimental diets, and management

The experimental work was conducted at the Small Ruminant Station of the Animal Science Research Institute of Iran, located in Karaj, Iran (35° 49′ 38″ N, 50° 56′ 56″ E), during the period spanning from May 2022 to August 2022. A total of fifty lactating Mahabadi goats, aged between 2 and 5 years, with an average body weight of 45.3 ± 7.20 kg and within 48 h post-parturition, were selected as subjects for this study. These goats were divided into two groups, carefully matched in terms of body weight (BW), days in milk (DIM), and milk yield (MY), and subsequently, they were randomly assigned to one of two distinct dietary treatments. The experiment was designed as a randomized complete block model, categorizing the two groups. Each group was accommodated in five replicates, with each replicate consisting of five goats. The study duration spanned 56 days, allowing for 21 days of diet acclimatization and dedicating 35 days for sample collection. To facilitate data collection, the dairy goats were individually placed in metabolic cages measuring 0.6 *m* × 1.2 m, beginning on the 15th day of the acclimatization period. Adequate measures were taken to ensure the animals' well-being. Two wet and dry bulb thermometers were strategically installed in the feeding barns, positioned at a height of 1.5 m above the ground, and the recorded average temperature throughout the experiment ranged between 18 °C to 22 °C. The goats were provided with a daily feed allowance totaling 1.8 kg of dry matter (DM) per goat. This approach allowed for controlled conditions and minimized exposure to environmental factors such as sun and rain. The goats individually had continuous access to water and were fed ad libitum with a total mixed ration (TMR) between 08:00 and 16:00 h daily. Dairy goats were milked twice daily, at 07:00 and 18:00. The experimental diets were formulated according to the recommendations of NRC (2007) and consisted of a control diet (C) without corn silage, and a diet including corn silage, which constituted 20 % of the diet's dry matter (DM). Tables 1 and 2 present the chemical composition of the diet ingredients and the experimental diets, respectively.

**Table 1:** Chemical composition of diet ingredients.

Ingredients	Chemical composition				
	Dry matter (%)	CP (g/kg DM)	ME (Mcal/kg DM)	Ca (g/kg DM)	P (g/kg DM)
Corn silage	25.91	81.00	2.32	6.10	1.90
Dry alfalfa	93.56	154.50	2.12	15.50	2.20
Wheat straw	95.00	37.80	1.50	3.90	0.80
Corn	89.86	94.00	3.46	2.40	2.80
Barley	91.97	110.00	3.57	2.00	3.10
Wheat bran	90.71	140.00	2.70	2.50	9.20
Soybean meal	91.17	433.00	3.78	6.60	7.10

**Table 2:** Experimental diets and their chemical composition.

Item	Experimental period	
	Control	Silage
Ingredients (g/kg DM)		
Corn silage	0.00	200.00
Dry alfalfa	130.00	80.00
Wheat straw	300.00	150.00
Corn	201.00	201.00
Barley	110.00	110.00
Soybean meal	70.00	70.00
Wheat bran	173.00	173.00
Calcium carbonate	8.00	8.00
Salt	3.00	3.00
Vitamin and mineral premix†	5.00	5.00
Chemical composition (%)		
Dry matter	92.44	78.70
Metabolizable energy (MJ/kg DM)	10.70	10.70
Crude protein	11.70	11.70
Non-fibrous carbohydrate	41.10	49.00
NDF	39.70	30.5
Crude fat	1.69	2.18
Phosphorous	0.36	0.38
Calcium	0.83	0.81
Ash	6.20	6.70

† Vitamin and mineral premix provided the following per kilogram of diet: cholecalciferol, 200,000 IU; vitamin A (from vitamin A acetate), 750,000 IU; vitamin E (from dl-a-tocopherol acetate), 4000 IU.; Na, 50 g; Mg, 20 g; Ca, 18 g; Zn, 17 g; Mn, 12 g; Fe, 6 g; Cu, 3.5 g; Co, 50 mg, I [from Ca (IO3)2•H2O], 150 mg, and Se, 10 mg.

### 2.3 Data collection

On days 0 and 56 of the experiment, individual weights of all animals were recorded at 8:00 AM after a 16-hour fasting period. Between days 50 and 52 of the experimental period, the daily feed intake of each goat was meticulously recorded. Subsequently, samples of the Total Mixed Ration (TMR) and any residues were collected and stored at −20 °C for later analysis. Concurrently, dairy milk yields from all the goats were documented during this period. Milk samples were systematically collected at two time points, 07:00 and 18:00, and then blended in a 1:1 ratio. A 50 mL subsample of milk was placed in vials treated with 2‑bromo-2-nitropropane-1–3-diol and preserved at 4 °C for compositional analysis. An additional 100 mL subsample, without any treatment, was preserved in two 50 mL centrifuge tubes at −20 °C. Fecal and urine samples were gathered daily from days 50 to 52 of the experimental period. Urine was collected in containers containing 200 mL of diluted sulfuric acid to maintain a pH level below 3. Urine volumes were documented daily, with 5 % of the urine samples stored in vials. Urine samples from each goat were pooled for three consecutive days and subsequently stored at −20 °C, awaiting nitrogen balance and purine derivative analysis. Fecal matter was collected under the metabolic cages in large metal nets, weighed daily, and approximately 5 % of each goat's feces were transferred into self-sealed plastic bags daily. These bags were mixed for three consecutive days and stored at −20 °C until chemical analysis was conducted. On days 53 and 54 of the experimental period, approximately one hour after the morning feeding, blood samples were drawn from the jugular vein using 10 mL vacutainer heparin-containing tubes. These tubes were promptly centrifuged at 3500 × g at 4 °C for 15 min to separate the plasma, following the procedure outlined by Alipanahi et al. (2019). Subsequently, the plasma samples were transferred to 2 mL centrifuge tubes for the assessment of plasma antioxidant capacity, biochemical parameters, and endocrine indices. On days 55 and 56, rumen fluid samples were collected through the ruminal cannula via suction using a hose 1 h after the morning feed (Mendowski et al., 2020). Rumen fluid was filtered through four layers of cheesecloth, and the pH was determined immediately using a mobile pH meter (Rocky Mount NC 27,894-USA). A 15 mL subsample of ruminal fluid was acidified immediately using 3 mL of metaphosphoric acid (25 %, m/v) to determine the volatile fatty acid content and ammonia-nitrogen (NH3-N) contents. A 40 mL subsample without metaphosphoric acid was collected into a 50 mL centrifuge tube and stored at –20 °C for ruminal microbial measurements analysis. All the samples of every goat and data point were included in the analysis.

### 2.4 Laboratory analysis

The chemical compositions of forage, concentrates, and diets were determined following the methods outlined in AOAC (2006). Specifically, dry matter (DM), ash, ether extract, crude protein, calcium, and phosphorus were analyzed using AOAC (2006) procedures, while acid detergent fiber (aNDF) was determined using the method described by Van Soest et al. (1991). Metabolizable energy was calculated based on the National Research Council (NRC, 2007) guidelines.

Milk samples were analyzed using an infrared spectroscopy technique (Milk Oscan, 134 BN Foss Electric, Hillerød, Denmark; AOAC, 2006). The analysis covered fat, non-fat solids, solids, crude protein, lactose, somatic cell counts, and pH. De novo fatty acids, free fatty acids, and mixed fatty acids in milk samples were measured following the procedures explained in Woolpert et al. (2016). Urea nitrogen concentration in milk was determined using a differential pH technique, as described by Luzzana and Giardino (1999). Milk lipids were extracted and methylated following the method of Folch et al. (1957), and the fatty acid profiles were assessed according to Feng et al. (2004). Milk beta-hydroxybutyrate, acetone, and non-esterified fatty acid content were measured using a clinical auto-analyzer (Hitachi 7020, Tokyo, Japan).

Plasma samples were analyzed for triglycerides, glucose, cholesterol, albumin, urea nitrogen, and total protein concentrations using a spectrophotometer (Genova; Jenway, Barloworld Scientific Ltd., Dunmow, Essex, UK) with commercial kits (Farasamed Diagnostics, Tehran, Iran). Ammonia content in rumen fluid was determined using a micro Kjeldahl apparatus (Kjeltec-UDK 126A), as described by Nasserian (1996).

Volatile fatty acids (VFA) in rumen fluid samples were provided, centrifuged (Mikro 220R, Hettich, Germany), and then analyzed using gas chromatography (Agilent 6890 Silica Capillary Column BPX-70) following the methods described in Bhandari et al. (2007) and Alipanahi et al. (2019).

The total antioxidant capacity of rumen liquor, plasma, and milk samples was measured using the ferric-reducing antioxidant power method, as explained by Benzie and Strain (1996)).

Urine samples previously stored at −20 °C were thawed, and allantoin, xanthine, hypoxanthine, and uric acid were extracted and evaluated using a spectrophotometer (Chen & Gomes, 1995). Allantoin was determined using the colorimetric method as explained by Chen and Gomes (1995), while uric acid concentration was measured using a commercial kit (U-9375, Sigma-Aldrich, Darmstadt, Germany). Xanthine and hypoxanthine contents were assessed after transformation to uric acid using the xanthine oxidase enzyme (Product No. X-1875, Sigma-Aldrich, Darmstadt, Germany).

Nitrogen balance was calculated based on the differences between total nitrogen intake and nitrogen excreted in urine, feces, and milk samples, following the method described by Philips and Rao (2001). The nitrogen concentrations in the samples of feed, urine, and feces were determined using a micro Kjeldahl apparatus, as outlined in AOAC (2006).

### 2.5 Statistical analysis

The Proc Mixed models in SAS 9.4 (SAS Institute Inc.) were used to evaluate experimental data. A randomized complete block design was used for data analysis. This experiment is carried out using the subsequent statistical model.

Y_ij_ = µ + T_i_ + B_j_ + e_ij_

where Y_ij_ is the value of each variable; µ is the mean of the related trait, T_i_ is the fix effect of corn silage or dry forage, B_j_ is the block effect, and e_ij_ error rate. Then LSD test method was used to compare the mean of each trait. Statistical significance was defined as *p* < 0.05.

## 3 Results and discussion

### 3.1 Performance and milk composition

Table 3 presents data on feed intake, milk composition, and yield in lactating Mahabadi goats fed a diet that included corn silage. The substitution of conventional dry forage with corn silage exhibited no statistically significant impact on feed intake, milk production, milk fat, solids, non-fat solids, protein, lactose content, total cell numbers, De novo relative fats, performed relative fats, and mixed relative fats percentages, as well as milk free fatty acid (FA) concentration, milk FA profile, β-hydroxybutyrate (BHBA), and acetone concentrations (*p* > 0.05). However, a noteworthy reduction in milk urea nitrogen concentration was observed (*p* = 0.03) in goats fed a diet containing corn silage. This finding contrasts with the results of a study by Khiang et al. (2015), which reported an increase in final body weight when Boer goats were fed corn silage instead of Napier grass. Nevertheless, in alignment with our observations, Canizares et al. (2011) found that substituting dry corn with high-moisture corn silage had no significant effect on milk composition in Alpine lactating goats. Similarly, a study on grazing dairy cows also reported a decrease in milk urea nitrogen when supplemented with corn silage (Dall-Orsoletta et al., 2020).

**Table 3:** Effect of substitution of dry forage with corn silage on performance, milk yield, and composition in lactating Mahabadi goats.

Item	Treatments		SEM	P-value
	Control	Corn silage		
Initial body weight (kg)	45.2	44.8	2.20	0.90
Final body weight (kg)	46.8	45.0	2.24	0.58
Dry matter intake (kg DM/day)	1706.7	1832.3	107.96	0.50
Milk yield (g/day)	795.1	876.8	109.5	0.60
Fat (%)	4.57	4.27	0.14	0.19
Protein (%)	4.58	4.49	0.11	0.54
Fat to protein ratio	1.13	1.20	0.04	0.19
Solids (%)	15.29	15.37	0.29	0.84
Non-fat solids(%)	10.02	9.97	0.09	0.69
Lactose (%)	4.57	4.61	0.04	0.56
Total cell numbers (× 10^3^ cell/mL)	1040.20	671.93	242.48	0.31
Urea nitrogen (mg/100 g)	15.48^a^	13.96^b^	0.45	0.03
*De novo* relative fats (%)	42.93	41.58	0.63	0.16
Preformed relative fats (%)	33.67	34.15	0.96	0.73
Mixed relative fats (%)	23.40	24.27	0.61	0.33
Free fatty acids (meq/100 g fat)	0.92	1.01	0.05	0.26
Saturated fatty acids (% total fatty acids)	79.51	78.37	2.08	0.54
Unsaturated fatty acids (%total fatty acids)	20.49	21.63	1.42	0.34
Monounsaturated fatty acids (%)	13.12	14.41	1.26	0.26
Polyunsaturated fatty acids (%)	7.37	7.22	0.26	0.68
C16:0 (%)	24.10	25.63	1.00	0.30
C18:0 (%)	11.05	11.30	0.44	0.69
C18:1C9 (%)	8.52	10.41	1.08	0.24
Non-esterified fatty acids (μeq/L)	480.58	530.91	33.42	0.31
Acetone (mmol/L)	0.22	0.21	0.01	0.38
Beta hydroxybutyrate (mmol/L)	0.12	0.13	0.01	0.44

^a,b^Items within row without common superscripts differ, *P* ≤ 0.05.

Urea, a minute organic compound, plays a pivotal role in assessing urinary nitrogen excretion and dietary nitrogen utilization efficiency. It originates in the liver through the conversion of ammonia, a byproduct resulting from the degradation of proteins and other nitrogenous molecules (Parker et al., 1995). Following its synthesis, urea rapidly enters the bloodstream and assumes the role of the primary nonprotein nitrogen constituent in milk (Broderick & Clayton, 1997). Both blood urea concentration (BUC) and milk urea concentration (MUC) have gained extensive utilization as nutritional markers in ruminants. This is largely due to their sensitivity to endogenous ammonia production, which is closely associated with gluconeogenesis and gastrointestinal tract activity (Hennessy & Nolan, 1988). Since urea serves as the principal endpoint in nitrogen metabolism for ruminants, the contents of urea in both blood and milk offer reliable indicators of nitrogen excretion (Zhai et al., 2005).

Numerous studies conducted on dairy cows have established a robust relationship between MUC and various dietary factors, including dietary crude protein intake (CPI), the proportion of protein subject to ruminal degradation and that which remains undegraded, as well as the protein-to-energy ratio in the diet (Butler et al., 1996; Oltner & Wiktorsson, 1983). Furthermore, due to their substantial impact on reproductive outcomes, there has been substantial research interest in the levels of blood urea nitrogen (BUN) and milk urea nitrogen (MUN) in animals (Kohn et al., 2005; Tshuma et al., 2014; Hamman et al., 2019). MUN is a valuable tool employed to assess the equilibrium of carbohydrate and nitrogen sources within the rumen environment, thereby providing critical insights into ruminal function (Jonker et al., 1999; Aguilar et al., 2012).

This balance in rumen digestion is vital not only for achieving optimal production in terms of quality and quantity but also for ensuring the health and well-being of the animals. Therefore, the decrease in milk urea nitrogen content observed when corn silage replaced dry alfalfa in the present experiment may be attributed to the synchronization between energy and protein sources within the rumen. This synchronization results in more efficient utilization of nitrogen, leading to reduced nitrogen excretion through milk and urine (Munyaneza et al., 2017). The protein-energy balance of the diet, the degradability of protein in the rumen, the availability of ammonia in the rumen, and the capacity of rumen microbes to capture it are factors that influence milk urea nitrogen content (Roseler et al., 1993). Thus, the lower milk urea nitrogen content in goats fed corn silage may indicate a more effective utilization of dietary nitrogen for milk production and body tissue synthesis.

### 3.2 Plasma biochemical parameters and metabolites

The incorporation of corn silage into the diet had no significant impact on the plasma levels of glucose, cholesterol, triglycerides, albumin, total protein, and the albumin to globulin ratio in lactating Mahabadi goats (Table 4) (*p* > 0.05). However, in goats fed a diet that included corn silage, a notable reduction in plasma urea nitrogen concentration was observed (*p* = 0.01). This observation aligns with the findings by Wang et al. (2016), which reported that the addition of corn silage to the diet had no influence on serum glucose and total protein concentrations but did increase serum triglyceride concentration. In contrast to our study, which identified a decrease in plasma urea nitrogen concentration when dry alfalfa/straw was substituted with corn silage, this trend in plasma urea nitrogen content is consistent with the results for milk urea nitrogen (Table 3) and may be attributed to the synchronous availability of both protein and energy in the rumen (Khaing et al., 2015).

**Table 4:** Effect of substitution of dry forage with corn silage on some plasma biochemical parameters in lactating Mahabadi goats.

Item	Treatments		SEM	P-value
	Control	Corn silage		
Urea nitrogen (mg/dl)	16.15^a^	14.20^b^	0.31	0.01
Glucose (mg/dl)	46.75	46.99	1.02	0.87
Cholesterol (mg/dl)	54.03	54.64	4.38	0.92
Triglyceride (mg/dl)	27.39	26.02	1.59	0.60
Albumin (g/dl)	2.43	2.36	0.16	0.75
Total protein (g/dl)	5.13	5.21	0.60	0.93
Albumin to globulin ratio	2.55	1.53	0.90	0.48

^a,b^Items within row without common superscripts differ, *P* ≤ 0.01.

In ruminants, a significant byproduct of the urea cycle is blood urea nitrogen (BUN), present in the serum or plasma components of the blood (Carlsson & Pehrson, 1993). Elevated BUN levels may serve as an indicator that the organism is not optimizing the utilization of dietary nitrogen (N) as efficiently as possible (Butler, 2005). To enhance production efficiency and minimize nitrogen losses in the environment, it is imperative to establish an equilibrium between dietary protein and energy (Hojman et al., 2004). The anticipated outcome of maintaining protein intake while elevating dietary energy levels is a reduction in BUN concentrations (Hammond, 1997). The inclusion of corn silage, renowned for its high starch content and energy provision, is likely a contributing factor to the observed decline in ruminal nitrogen concentration. Nevertheless, it is noteworthy that Wang et al. (2016) reported no significant impact on blood urea nitrogen concentration when lactating goats were provided with a diet containing corn silage.

### 3.3 Nitrogen balance and urinary purine derivatives

Table 5 presents the influence of substituting conventional dry forage with corn silage on total purine derivatives and nitrogen balance. Notably, none of the assessed parameters exhibited significant differences (*p* > 0.05). The dietary replacement of dry forage with corn silage did not have a statistically significant effect on nitrogen balance and urinary purine derivatives. It's essential to acknowledge the findings of Al-Marashdeh et al. (2015), who observed reduced urinary nitrogen excretion in dairy cows fed corn silage compared to those on herbages. The excreted purine derivatives are recognized as indicators of the ruminal outflow of microbial protein Tas and Susenbeth (2007). The utilization of nitrogen is intricately tied to the processes of nitrogen excretion and retention, which stand as pivotal indicators of nitrogen metabolism and are critical for ascertaining the nutritional status of ruminants, particularly concerning protein Firkins et al. (2007). Notably, heightened nitrogen retention within the animal's body is a characteristic of species that consume substantial quantities of dietary protein. Sarwar et al. (2003) have demonstrated that both the presence of fermentable carbohydrates in the diet and the quantity of nitrogen ingested play integral roles in enabling the organism to retain nitrogen efficiently. Additionally, urinary purine derivatives (PDs) are widely acknowledged as crucial variables for assessment, primarily because rumen microorganisms constitute the primary source of dietary protein for ruminants (Chen et al., 1992). Thus, microbial protein production can be estimated based on the urinary excretion of PDs, which primarily include allantoin, uric acid, xanthine, and hypoxanthine, and are predominantly of microbial origin Salman et al. (2013); Ma et al. (2014).

**Table 5:** Effect of substitution of dry forage with corn silage on urine purine derivatives, microbial nitrogen, and nitrogen balance in lactating Mahabadi goats.

Item	Treatments		SEM	P-value
	Control	Corn silage		
Total purine derivatives (mmol/L)	13.8	14.1	3.30	0.20
The absorbed purine derivatives (mmol/L)	1.86	1.98	0.23	0.14
The excreted purine derivatives (mmol/L)	2.08	2.11	0.20	0.12
Nitrogen intake (g/d)	29.0	30.9	0.76	0.39
Milk nitrogen (g/d)	5.00	6.60	0.45	0.45
Feces nitrogen (g/d)	14.1	12.0	0.54	0.38
Urine nitrogen (g/d)	6.90	7.80	0.19	0.11
Microbial nitrogen (g/d)	8.45	9.71	0.77	0.11
Nitrogen retention (g/d)	3.00	4.50	0.39	0.15
Creatinine (mmol/L)	0.88	0.98	0.10	0.10

The quantification of purine derivatives (PDs) excreted in urine serves as a reliable indicator for measuring microbial protein production within the rumen. This methodology relies on the premise that duodenal purine bases are well-absorbed, with their derivatives primarily eliminated through renal excretion, rendering them dependable microbiological markers. As posited by Belenguer et al. (2002), the ratio of PD in urine can be employed to forecast the flow of microbial nitrogen. Notably, a considerable proportion of PDs found in ruminant urine results from the incomplete digestion of microbial nucleic acid entering the duodenum. However, in the context of the current study, it is noteworthy that no statistically significant differences in urine PDs were observed between diets. Furthermore, no distinctions were evident in the excretion of uric acid and hypoxanthine among the corn-based diets. Intriguingly, the efficiency of microbial nitrogen production remained unaltered by the dietary interventions explored in this investigation. A multitude of factors, including the quantity and sources of nitrogen and carbohydrates, may contribute to the constancy of urine purine derivatives (PDs) (Singh et al., 2007). In both dietary scenarios under examination, it is apparent that the energy and protein content of the diets are sufficient to sustain optimal microbiological growth. Furthermore, the urine samples affirm the goats' capability to oxidize ingested purine bases into non-utilizable purine derivatives (PDs).

### 3.4 Rumen fermentation parameters

The impact of dietary inclusion of corn silage on rumen parameters, such as the concentration of individual volatile fatty acids (VFA), pH, and NH3, is detailed in Table 6. Goats fed a diet containing corn silage exhibited a lower rumen pH before feeding (*p* = 0.02) and tended to have a lower rumen ammonia-N concentration three hours after feeding (*p* = 0.07). These results concur with the observations made by Lettat et al. (2013), who noted a reduction in rumen pH in dairy cows when alfalfa was replaced with corn silage. The higher NDF concentration in the control diet likely contributed to its greater rumen buffering effect, which is associated with the higher water-holding capacity of the digest (Kmicikewycz & Heinrichs, 2015). The ruminal pH plays a crucial role in dry feed matter digestibility (DMD) and the survival and development of protozoa (ciliates) (Song et al., 2021). Numerous studies have consistently highlighted the deleterious impact of low pH levels on key nutrient factors, such as protein degradability (Hu et al., 2004; Cantalapiedra-Hijar et al., 2011; Guo et al., 2021). The adverse effects of low pH on microbial fermentation have been attributed to the magnitude of the pH reduction (Cerrato-Sánchez et al., 2008).

**Table 6:** Effect of substitution of dry forage with corn silage on ruminal fermentation parameters in lactating Mahabadi goats.

Item	Treatments		SEM	P-value
	Control	Corn silage		
Before feeding				
pH	7.05^a^	6.72^b^	0.09	0.02
Ammonia nitrogen (mg/dl)	17.96	18.96	0.97	0.11
Total volatile fatty acids (mmol/L)	58.24	73.80	6.44	0.13
Acetic acid (%)	60.99	62.00	1.27	0.58
Propionic acid (%)	18.83	15.96	1.44	0.21
Butyric acid (%)	11.75	12.97	0.90	0.37
Isovaleric acid (%)	2.90	3.20	0.28	0.46
Valeric acid (%)	2.54	2.42	0.21	0.69
Isobutyric acid (%)	2.99	3.45	0.31	0.79
Acetic acid to propionic acid ratio	3.56	4.05	0.33	0.31
Three hours after feeding				
pH	6.34	6.21	0.09	0.32
Ammonia nitrogen (mg/dl)	21.26	17.01	1.51	0.07
Total volatile fatty acids (mmol/L)	95.60	102.45	6.85	0.51
Acetic acid (%)	56.39	54.69	1.38	0.42
Propionic acid (%)	26.01	26.83	2.08	0.79
Butyric acid (%)	12.06	13.33	0.91	0.33
Isovaleric acid (%)	1.51	1.46	0.16	0.83
Valeric acid (%)	2.63	2.34	0.20	0.33
Isobutyric acid (%)	1.40	1.35	0.15	0.80
Acetic acid to propionic acid ratio	2.39	2.13	0.24	0.48

^a,b^Items within row without common superscripts differ, *P* ≤ 0.05.

Further underscoring the connection between low pH and reduced ruminal degradability, Farenzena et al. (2014) reported diminished ruminal degradability at low pH levels. This decrease was linked to a decline in bacterial adherence to the substrate, while the activity of fibrolytic enzymes remained unaltered. Moreover, a drop in rumen pH is associated with a decline in the generation of short-chain fatty acids, primarily acetate, along with reduced feed digestibility and daily fermentation gas output. These findings collectively emphasize the negative repercussions of low pH on critical aspects of nutrient utilization and microbial fermentation in ruminants. Furthermore, the control diet, containing dry forage, might have stimulated increased chewing activity and, subsequently, elevated saliva production, resulting in a higher rumen buffering capacity and higher pH levels.

However, in our study, the substitution of dry forage with corn silage did not significantly influence total ruminal VFA, individual VFA concentrations, and acetic acid to propionic acid ratio. In contrast, Lettat et al. (2013) reported a decrease in acetate and butyrate concentrations and an increase in propionate concentration when alfalfa was replaced with corn silage in the diet of dairy cows. Since at least the 1940s, the concentrations of ruminal volatile fatty acids (VFA) and other analytes have served as pivotal tools for characterizing the dynamics of in vivo ruminal fermentation and the consequences of dietary interventions (Hall, 2015). The intricate interplay between fermentable energy and microbial protein synthesis within the rumen fosters the production of volatile fatty acids (VFA) from both structural and non-structural carbohydrates (Orskov, 1992). This dynamic interaction yields a range of effects on ruminants, primarily due to variations in energy retention efficiency associated with different carbohydrate sources. It is particularly noteworthy that this efficiency appears to exhibit a negative correlation with the molar proportion of acetic acid generated in the rumen, a relationship more pronounced in high-fiber diets. This phenomenon underscores the central role of nitrogen (N) in ruminal fermentation processes, as it is an indispensable resource for the survival of rumen microorganisms (Belanche et al., 2012).

Interestingly, goats fed a diet containing corn silage exhibited lower ruminal ammonia nitrogen content three hours after feeding. This reduction can be attributed to the synchronized availability of energy substrates (corn silage, a rich source of starch) and nitrogen components, leading to higher microbial protein production (Broderick, 2003). Furthermore, high-starch diets (containing corn silage) are speculated to decrease protozoa numbers (Martin et al., 2010), potentially contributing to the reduction in ruminal ammonia levels. The elimination of protozoa could result in decreased bacterial protein breakdown and feed protein degradability in the absence of rumen protozoa. The observed decrease in rumen ammonia in our study is consistent with the findings of Benchar et al. (2014) in lactating dairy cows fed corn silage.

### 3.5 Antioxidant capacity

Table 7 presents the effects of dietary inclusion of corn silage on the antioxidant potential in lactating Mahabadi goats. Goats receiving a diet containing corn silage exhibited significantly higher levels of antioxidant capacity in rumen fluid, plasma, and milk when compared to goats fed a dry forage diet (*p* < 0.05). This enhancement can be attributed to the presence of anthocyanin, a polyphenol extracted from corn silage, known for its antioxidant activity (Kim et al., 2023). Anthocyanin contributes to the body's antioxidant capacity by donating phenolic hydrogen atoms to free radicals and enhancing the activity of antioxidant-related enzymes. The increase in plasma antioxidant function in sheep fed corn silage has been reported by Hosoda et al. (2012), and Matsuba et al. (2019) observed a rise in blood superoxide dismutase concentration with the dietary addition of corn silage in dairy cows.

**Table 7:** Effect of substitution of dry forage with corn silage on the antioxidant potential of rumen liquor, plasma, and milk in lactating Mahabadi goats.

Antioxidant capacity (mmol Fe+2/L)	Treatments		SEM	P-value
	Control	Corn silage		
Rumen liquor	1.76^b^	1.96^a^	0.06	0.03
Plasma	0.28^b^	0.32^a^	0.08	0.01
Milk	1.38^b^	1.72^a^	0.05	0.01

^a,b^Items within row without common superscripts differ, *P* ≤ 0.05.

It is crucial to note that among animals, the two primary antioxidant enzymes are catalase and superoxide dismutase (SOD). As highlighted by Tsugami et al. (2017), serum antioxidant activity constitutes a vital component of the oxidative defense mechanisms in animals, serving as an indicator of oxidative stress. Notably, research has elucidated that the inclusion of flavonoids in the diet, such as those found in corn silage, can augment the antioxidant capacity in poultry and cattle, akin to their effect in humans. For instance, hens supplemented with soybean isoflavones exhibited elevated plasma levels of superoxide dismutase (SOD) and total antioxidant capacity (T-AOC (Jiang et al., 2007).

Furthermore, supplementing broiler diets with flavonoids derived from Scutellaria baicalensis Georgi improved their resilience against oxidative stress (Liao et al., 2018), while dairy cows fed forage flavone extract exhibited increased activities of both glutathione peroxidase (GSH-Px) and SOD (Zhan et al., 2017). These findings are in line with the outcomes reported by Zhong et al. (Zhong et al., 2015) in skeletal muscle cells from goats subjected to Camellia sinensis supplementation in vitro.

Additionally, these findings imply that these antioxidants can be useful in postponing the onset of off-flavors and discolouration in meat, improving the quality and shelf life of meat products (Pateiro et al., 2018).

### 3.6 Milk composition and FA profile during different weeks of lactation

Table 8 shows the interaction effect of treatment (diet) and week of lactation on milk composition from two to eight weeks of lactation. The interaction effect of treatment (diet) and week of lactation and the diet effect did not significantly influence milk compositions (*p*>0.05). However, the effect of the week was significant, and milk protein percentage, non-fat solids, and performed relative fats had the highest content during the eighth week of lactation. The percentage of lactose and milk urea nitrogen had the greatest value during the sixth week of lactation. The interaction effect of diet and week of lactation on milk FA profile and ketone body concentrations is presented in Table 9. The interaction effect of diet and the week of lactation, as well as the diet effect, had no significant impact on milk FA profile and ketone bodies (*p* > 0.05). However, the effect of the week was significant, and milk unsaturated fatty acids, monounsaturated fatty acids, polyunsaturated fatty acids, C16:0, C18:0, and C18:1C9 had the highest percentages during the second week of lactation, while milk saturated fatty acids had the lowest percentages during the second week of lactation. Milk BHBA and acetone concentrations had the highest content during the fourth and the sixth week of lactation, respectively, while milk NEFA concentration had the lowest content during the sixth week of lactation. The variation in milk compositions during different weeks of lactation is consistent with previous studies. The higher milk fat-to-protein ratio observed in the sixth week of lactation may be attributed to adipose tissue mobilization and an enhanced supply of fatty acids for milk fat synthesis (Kuczyńska et al., 2021). El-Tarabany et al. (2018) reported no significant changes in milk protein, fat, and SNF percentages during the first 80 days of lactation in Baladi goats. Additionally, Strzałkowska et al. (2009) reported an increase in protein content with the progress of lactation, and Ibnelbachyr et al. (2015) showed higher lactose contents in the early and late stages of lactation. The increase in C18:0 and decrease in C18:1 cis-9 with advancing lactation observed in our study align with earlier findings (Stoop et al., 2009). A negative energy balance during early lactation may lead to significant mobilization of body fat in high-producing dairy cows (Palmquist et al., 1993). Additionally, Jorjong et al. (2014) suggested that the increment in milk C18:1 cis-9 concentration during the second week of lactation may play an essential role in early alerting to a risk of detrimentally high blood NEFA concentration. Further research is warranted to elucidate the underlying mechanisms behind the variations in milk composition and fatty acid profiles throughout different stages of lactation in response to dietary interventions.

**Table 8:** Effect of substitution of dry forage with corn silage on milk composition during weeks of lactation in lactating Mahabadi goats.

Milk analysis	Treatments		Lactation week				SEM	P-value		
	Control	Corn silage	2	4	6	8		Treat	Week	Treat*Week
Fat (%)	4.27	4.56	4.31	4.19	4.67	4.50	0.16	0.21	0.41	0.55
Protein (%)	4.58	4.49	4.68^ab^	4.35^bc^	4.12^c^	4.99^a^	0.12	0.57	<0.01	0.68
Fat to protein ratio	0.95	1.02	0.94^b^	0.96^b^	1.14^a^	0.92^b^	0.02	0.05	<0.01	0.87
Lactose (%)	4.57	4.61	4.48^b^	4.49^b^	4.73^a^	4.67^ab^	0.04	0.57	0.02	0.82
Solids (%)	15.29	15.37	16.13	15.54	15.16	15.48	0.34	0.86	0.06	0.81
Solids nonfat (%)	10.02	9.97	9.99^b^	9.72^b^	9.76^b^	10.52^a^	0.10	0.70	<0.01	0.64
Total cell numbers (× 10^3^ cell/ml)	1040.18	671.93	248.50	978.80	1200.70	996.20	258.59	0.33	0.30	0.57
Urea nitrogen (mg/100 g)	13.96	15.48	13.31^b^	16.87^a^	16.47^a^	12.24^b^	0.57	0.08	<0.01	0.23
*De novo* relative fats (%)	42.93	41.58	40.99^b^	41.08^b^	43.59^a^	43.35^ab^	0.57	0.11	0.04	0.09
Mixed relative fats (%)	23.40	24.27	24.76^a^	25.76^a^	25.06^a^	19.76^b^	0.67	0.37	<0.01	0.53
Preformed relative fats (%)	33.67	34.15	34.24^ab^	33.16^b^	31.35^b^	36.89^a^	0.95	0.73	<0.01	0.27
Free fatty acids (meq/100 g fat)	0.92	1.01	1.17^a^	1.28^a^	0.83^b^	0.57^b^	0.06	0.33	<0.01	0.98

^a-c^Items within row without common superscripts differ, *P* ≤ 0.01.

^a,b^Items within row without common superscripts differ, *P* ≤ 0.05.

**Table 9:** Effect of substitution of dry forage with corn silage on milk fatty acids profile and serum ketone bodies concentrations during weeks of lactation.

Milk analysis	Treatments		Weeks after lactation				SEM	P-value		
	Control	Corn silage	2	4	6	8		Treat	Week	Treat*Week
Saturated fatty acids (%)	79.51	81.37	65.77^b^	78.17^a^	74.22^a^	73.60^a^	2.16	0.55	<0.01	0.57
Unsaturated fatty acids (%)	20.92	22.89	28.93^a^	20.31^b^	19.10^b^	19.27^b^	1.35	0.31	<0.01	0.26
Monounsaturated fatty acids (%)	13.89	15.97	20.94^a^	14.68^b^	12.96^b^	11.15^b^	1.16	0.22	<0.01	0.23
Polyunsaturated fatty acids (%)	7.37	7.22	8.45^a^	5.90^b^	6.46^b^	8.37^a^	0.31	0.73	<0.01	0.57
C16:0 (%)	24.10	25.62	31.15^a^	26.00^b^	23.09^b^	19.22^c^	0.96	0.28	<0.01	0.51
C18:0 (%)	11.05	11.30	12.08^a^	8.92^b^	10.27^b^	13.43^a^	0.46	0.70	<0.01	0.44
C18:1C9 (%)	8.52	10.41	15.30^a^	10.85^b^	7.10^bc^	4.62^c^	0.98	0.19	<0.01	0.19
Non-esterified fatty acids (μeq/L)	480.58	530.91	491.06^a^	566.51^a^	346.28^b^	619.13^a^	33.43	0.30	<0.01	0.03
Beta hydroxybutyrate (mmol/L)	0.12	0.13	0.12^b^	0.18^a^	0.11^bc^	0.09^c^	0.009	0.47	<0.01	0.95
Acetone (mmol/L)	0.22	0.21	0.16^c^	0.14^c^	0.31^a^	0.25^b^	0.01	0.36	<0.01	0.83

^a-c^Items within row without common superscripts differ, *P* ≤ 0.01.

## 4 Conclusions

Overall, the dietary inclusion of corn silage as a substitute for conventional dry forage such as dry alfalfa showed no significant differences in performance, milk composition, and nitrogen balance in Mahabadi lactating goats. Consequently, the utilization of corn silage is proposed due to its economic advantages, especially in regions with limited access to dry forage, such as arid and semi-arid conditions. Moreover, corn silage exerted a positive influence on rumen fermentation, antioxidant capacity, and milk fatty acid profiles. These findings offer valuable insights into the potential benefits of incorporating corn silage as a forage substitute in the diet at a concentration of 200 g/kg DM per lactating goat. This research contributes to expanding our existing knowledge and provides additional dietary recommendations for dairy goats.

## Funding

Not Applicable.

## Ethical statement

The authors verify that the ethical policies of the journal, as explained on the journal's author guidelines page, have been adhered to, and the proper ethical review committee approval has been obtained. The authors verify that they have followed EU standards for protecting animals used for scientific purposes (Protocol No. 2022–015A).

## Data availability

The data that support the findings of this study are available upon reasonable request.

## Author contributions

Sh.T.S. and A.F. conceived the ideas. Sh.T.S., A.F. and N.P. designed the methodology. Sh.T.S. and A.F. analyzed the data. Sh.T.S., A.F., N.P. and S.R.E.M. interpreted the data. N.P. and S.R.E.M. validated the data. Sh.T.S. wrote the main manuscript. A.F., N.P. and S.R.E.M. reviewed and edited the manuscript. All authors read and approved the final version of manuscript.

## Declaration of Competing Interest

The authors declare that they have no known competing financial interests or personal relationships that could have appeared to influence the work reported in this paper.
